# Symptom-Based Assessment and Advice to Seek Medical Attention by Community Pharmacists: A Case Series

**DOI:** 10.7759/cureus.97982

**Published:** 2025-11-27

**Authors:** Yasunari Kinoshita

**Affiliations:** 1 Community Pharmacy, Welcia Pharmacy Co. Ltd., Tokyo, JPN

**Keywords:** community pharmacy, evidence-based clinical practice, japan, lqqtsfa, ‘self-medication’, symptom-based assessment, triage

## Abstract

Community pharmacists increasingly serve as the first point of contact in primary healthcare as self-medication and over-the-counter (OTC) drug use expand, yet structured methods for symptom assessment and referral remain underdeveloped in Japan. This case series describes three patients in whom pharmacists used the LQQTSFA framework (Location, Quality, Quantity, Timing, Setting, Factors, Associated symptoms) to organize symptoms within current regulations and advise on seeking medical care. The cases involved chronic headache, laxative-induced diarrhea, and anxiety-related frequent consultations. In each case, pharmacists used structured questioning to clarify the clinical significance of the complaint, reframed patients’ self-assessments, and cross-referenced findings with relevant clinical guidelines, leading to appropriate medical referrals that resulted in accurate physician diagnoses and clinical improvement. Across cases, a reproducible three-step pattern emerged: structuring the chief complaint, validating decisions with evidence-based references, and supporting behavioral change through follow-up. These cases suggest that structured symptom-based assessments by community pharmacists can promote early, safe, and rational healthcare-seeking without encroaching on diagnostic authority and may provide a practical foundation for educational and systemic implementation in Japan’s evolving primary care.

## Introduction

In recent years, the promotion of self-medication and the widespread availability of over-the-counter (OTC) medicines have strengthened the role of community pharmacies as the first point of contact for residents seeking medical advice [1]. With the establishment of the registered sales clerk system and the progress of digitalization, an increasing number of patients continue to use OTC drugs without the involvement of pharmacists. Consequently, issues such as duplicate medication, adverse drug reactions, off-label use, overdosing, and other forms of medication misuse have become more apparent [1,2]. Under these circumstances, it is critically important for community pharmacists to accurately assess patients’ symptoms at an early stage and to recommend medical consultation when appropriate in order to maintain the quality and safety of community healthcare.

However, in Japan, no standardized method has yet been established for pharmacists to systematically organize patients’ chief complaints and link them to clinical decisions regarding medical referral. Existing studies have largely been limited to surveys on current listening practices, with few reports that visualize the reasoning processes underlying pharmacists’ clinical judgments [3]. The revised 2021 Model Core Curriculum for Pharmacy Education emphasizes the cultivation of clinical reasoning skills based on symptomatology; however, evidence connecting such education to practical implementation remains limited [4]. LQQTSFA (Location, Quality, Quantity, Timing, Setting, Factors, Associated symptoms) is a structured questioning framework widely used in medical interviews and clinical reasoning education. It enables multidimensional organization of symptoms, providing the foundation for differential diagnosis and severity assessment [3]. Applying the LQQTSFA method to community pharmacy practice may allow pharmacists to perform symptom-based assessments without encroaching upon diagnostic authority, thereby enhancing the validity of their referral decisions.

Meanwhile, in countries such as the United Kingdom and the United States, systems like “Pharmacy First” and Collaborative Drug Therapy Management (CDTM) have institutionalized pharmacists’ roles in primary response and triage [5-7]. In contrast, Japan’s current legal framework restricts pharmacists’ diagnostic and prescribing authority, often limiting referral activities to formal advice without structured clinical reasoning [1,8]. Therefore, it is necessary to elucidate how community pharmacists in Japan currently exercise clinical judgment within these institutional constraints and how they facilitate appropriate medical referrals. The present study aims to explore and describe the process by which community pharmacists conducted symptom-based assessments using the LQQTSFA framework to support referral decisions in three real-world cases. By comparing multiple cases, this study seeks to extract common reasoning patterns and propose a reproducible scheme for symptom-based assessment and patient referral conducted by community pharmacists.

## Case presentation

Case 1

A woman in her 40s visited the pharmacy with a chief complaint of recurrent headaches that had persisted for several years. She had no notable medical history and had been continuously using over-the-counter (OTC) analgesics (NSAIDs) on her own judgment to treat her chronic headaches. The community pharmacist performed a symptom-based assessment based on the LQQTSFA framework, and the following findings were obtained: The headache was localized in the temporal region (location) and was characterized by throbbing pain (quality). Although the symptoms temporarily improved with the use of NSAIDs, they did not completely disappear and recurred repeatedly (quantity). The headache persisted chronically but tended to worsen during the menstrual cycle or when barometric pressure dropped (timing). Nausea and visual aura (scintillating scotoma) were also present, and the symptoms interfered with her daily activities (setting, associated symptoms).

Based on these findings, the pharmacist compared the characteristics and associated symptoms of the headache with the typical features of migraine described in the Clinical Practice Guideline for Headache 2021 [9] and the International Classification of Headache Disorders, 3rd edition (ICHD-3) [10]. It was determined that continued self-medication with OTC drugs would be insufficient, and the pharmacist advised that a detailed medical evaluation by a physician was necessary, leading to a referral recommendation. At that time, in order to objectively understand the severity of symptoms and their impact on daily life, the pharmacist asked the patient to complete the Headache Impact Test (HIT-6) [11] self-assessment, which resulted in a score of 67 points. After visiting a medical institution, the patient was definitively diagnosed with refractory migraine, and treatment with a calcitonin gene-related peptide (CGRP) antibody was initiated. Three months after treatment, the frequency of headache attacks decreased, and the HIT-6 score improved from 67 to 63. The patient stated, “Understanding the characteristics of my headache has reduced my anxiety,” and the frequency of self-directed analgesic use also decreased.

This case demonstrates an example in which a community pharmacist enhanced the appropriateness of a medical referral by combining symptom-based assessment with guideline reference. Without engaging in diagnostic activity, the pharmacist promoted appropriate consultation behavior through the structuring of symptoms and the utilization of existing medical knowledge, showing both educational and practical significance.

Case 2

A man in his seventies presented to the pharmacy with a chief complaint of constipation. He had a history of cardiovascular disease and was regularly prescribed antiplatelet agents (aspirin/lansoprazole combination and dipyridamole) as well as laxatives (elobixibat and macrogol). In addition to these prescribed medications, he had been continuously taking over-the-counter (OTC) magnesium hydroxide products on his own judgment. Because of the excessive number of medications, the pharmacist suspected polypharmacy and possible OTC drug overuse and conducted a structured interview based on the LQQTSFA framework to assess the actual situation. The abdominal symptoms extended throughout the abdomen (location), and frequent discharge of soft to watery stools was observed (quality). The patient reported having three to four bowel movements per day, indicating that what he perceived as “constipation” was, in fact, a diarrheal bowel dysfunction (quantity). The symptoms worsened, particularly after taking magnesium hydroxide (timing), and anxiety about bowel movements had led to habitual additional use of OTC drugs (setting). A typical pattern of exacerbation and remission was observed, with improvement after discontinuing magnesium hydroxide (Factors). The patient also complained of abdominal discomfort as an associated symptom (associated symptoms).

Based on the results of the symptom-based assessment, the community pharmacist referred to the Clinical Practice Guideline for Chronic Constipation 2023 [12] and used the Bristol Stool Form Scale (BSFS) as a communication aid to help the patient accurately understand and describe stool consistency [13]. These references clarified that the patient’s complaint of “constipation” was actually a low-residue diarrheal condition caused by excessive laxative use, resulting in frequent watery stools and a depleted intestinal content. The pharmacist explained the potential adverse effects of continued magnesium hydroxide use, such as hemorrhoids and electrolyte imbalance, and recommended discontinuation. In addition, the patient was advised to use macrogol within the appropriate dosage range, as there was a tendency toward overuse. These interventions were summarized in a tracing report and shared with the attending physician, allowing for collaborative optimization of pharmacotherapy.

At a two-month follow-up, the patient had discontinued the additional OTC medications and continued macrogol at the prescribed dose. The frequency of bowel movements stabilized at once per day, with stools of medium-soft consistency, and abdominal discomfort resolved. This case demonstrates how a community pharmacist, by using symptom-based evaluation together with relevant clinical references, was able to enhance clinical judgment and contribute to the optimization of pharmacotherapy. The combination of structured assessment and reference to established medical information suggests that pharmacist-led support at the community level can address drug-related bowel dysfunction and related psychological factors.

Case 3

A man in his sixties had frequently visited multiple medical departments, including otolaryngology, dermatology, and urology, over the past two years. He was a regular user of the same community pharmacy and reacted excessively to minor physical discomfort or nonspecific symptoms, visiting or calling the pharmacy up to ten times a day. Even after medication counseling, he continued to ask questions such as “Is this medicine really safe?” or “Could you explain it again?”, which interfered with pharmacy operations. The community pharmacist suspected that an underlying clinical factor might be contributing to the patient’s persistent anxiety and conducted a structured interview based on the LQQTSFA framework. No clear physical symptoms were observed (location), but the patient showed marked anxiety and fear in response to minor bodily sensations (quality). Temporary reassurance was achieved after counseling, but anxiety reappeared within a few hours, prompting repeated visits and phone calls (quantity, timing). Frequent contact occurred regardless of the time of day (setting), and a psychological pattern of transient reassurance followed by recurrence of anxiety was observed (factors). The patient also exhibited restlessness and repeatedly asked the same questions (associated symptoms).

Based on these findings, the pharmacist considered that the patient’s behavior might be influenced by an underlying anxiety-related clinical factor rather than simple excessive worry. The NICE guideline for Generalised Anxiety Disorder and Panic Disorder (CG113) [14] recommends professional therapeutic intervention when anxiety and repetitive checking behaviors significantly impair daily functioning. This evidence was used to support the pharmacist’s reasoning, and the patient was reassured without criticism of his personality. A psychiatric consultation was gently recommended. Subsequently, the patient visited a psychiatric clinic and was diagnosed with anxiety neurosis. He was first prescribed escitalopram 10 mg, but due to drowsiness, the treatment was switched to vortioxetine 10 mg. The pharmacist continued follow-up to monitor adherence and side effects.

Before the intervention, the patient had been visiting or calling the pharmacy up to ten times a day, corresponding to an estimated total of around 200 contacts per month, based on consistent recollection among multiple pharmacy staff members. One month after psychiatric treatment began, the frequency decreased to one or two times per month. The patient and his family reported that calmness and peace of mind had returned, and the sense of restlessness subsided. Approximately one year later, the psychiatrist confirmed remission, and treatment was completed. This case illustrates how a community pharmacist can reassess daily consultation behaviors from a symptom-based perspective, infer psychological contributing factors, and facilitate appropriate referral to a specialist. Using structured questioning and established clinical guidelines, the pharmacist contributed to timely therapeutic intervention and improvement in the patient’s quality of life (QOL) without engaging in diagnostic activity. 

Table 1 shows the summary of all three cases.

**Table 1:** Summary of key findings and LQQTSFA-based assessment This table summarizes the main clinical features, LQQTSFA assessment items, and referral rationales for Cases 1–3. All information is derived from patient interviews conducted by community pharmacists, without involving diagnostic procedures.

Item	Case 1	Case 2	Case 3
Chief complaint	Chronic headache	Constipation (self-reported)	Repetitive reassurance-seeking
Medications involved	OTC NSAIDs	Laxatives + OTC Mg(OH)₂	None significant
L: Location	Temporal/cranial	Generalized abdomen	No physical location
Q: Quality	Throbbing	Soft to watery stools	Anxiety toward minor sensations
Q: Quantity	Frequent/refractory	3–4 bowel movements/day	Up to 10 contacts/day
T: Timing	Persistent despite OTC	Worse after Mg(OH)₂	Anxiety recurs within hours
S: Setting	Daily activity triggers	Habitual OTC use	Anytime, frequent contact
F: Factors	NSAID overuse worsened	Laxative excess → diarrhea	Reassurance–anxiety cycle
A: Associated symptoms	Nausea, aura	Abdominal discomfort	Restlessness, repeated questions
Guideline referenced	Headache GL	Chronic Constipation 2023	NICE (GAD/panic)
Pharmacist action	Referral → CGRP-mAb	Stop Mg(OH)₂; optimize macrogol	Psychiatric referral
Outcome	Improved	Bowel rhythm normalized	Marked reduction; remission

## Discussion

In all three cases, the pharmacists systematically organized the patients’ complaints in accordance with the elements of the LQQTSFA framework, clarifying the location, quality, course, and contributing factors of each symptom. This structured approach enabled accurate characterization of the clinical picture without relying on the patients’ own interpretations or exaggerated descriptions.

In Case 1, persistent headaches managed with long-term self-medication were found to align with features of migraine requiring professional evaluation. In Case 2, the patient’s complaint of “constipation” was revealed to be drug-induced diarrhea caused by excessive laxative use. In Case 3, repeated reassurance-seeking triggered by minor bodily sensations was reinterpreted as behavior influenced by an underlying anxiety-related condition.

These findings support the role of symptom-based assessment not merely as information gathering, but as an initial form of clinical reasoning that helps determine the necessity of medical evaluation.

The pharmacists further strengthened their decision-making by cross-checking their initial impressions against relevant clinical guidelines, including those for headache, chronic constipation, and anxiety-related conditions. These guidelines were not used for diagnostic purposes but to contextualize patient information within established medical knowledge, thereby enabling evidence-based pre-medical evaluation.

This process contributed to more objective and reproducible assessments, independent of individual experience, and enhanced the appropriateness of referral decisions. Matsushita et al. reported similar findings in Japan, noting that structured interviewing and clinical-reasoning training improved pharmacists’ ability to collect patient information and make consistent triage decisions in community settings [15].

Improvements following appropriate medical intervention were observed in all three cases. In Case 1, initiation of CGRP-targeted therapy reduced headache frequency and improved quality of life. In Case 2, discontinuation of excessive laxatives and optimization of macrogol therapy stabilized bowel habits. In Case 3, psychiatric referral and subsequent treatment markedly reduced reassurance-seeking behavior, improving daily functioning for the patient and family.

These outcomes indicate that structured symptom assessment in community pharmacies can effectively guide patients toward appropriate medical care and contribute to improved clinical outcomes.

Pharmacy interviewing practices in Japan have historically relied heavily on individual pharmacists’ experience, often lacking a systematic structure. The consistent workflow observed in these three cases: (1) structured symptom organization, (2) validation of impressions against evidence, and (3) facilitation of referral and follow-up, resembles what Matsushita described as the need for pharmacists to adopt standardized clinical-reasoning models within community-based integrated care [15]. This sequence does not constitute a diagnosis but represents a form of “pre-diagnostic clinical reasoning” that remains within the pharmacist’s legal scope of practice. Its reproducible application across cases with different health problems suggests potential value for pharmacy education and daily clinical practice.

In this case series, pre-diagnostic clinical reasoning refers to the structured organization of symptoms and the verification of clinical impressions using established clinical guidelines without engaging in diagnostic activities. This approach has gained increasing attention in pharmacy education and community-based care in Japan, where pharmacists are expected to use structured questioning frameworks to support safe and timely referral within the limits of their professional role.

This report includes a small number of cases and may be subject to selection bias. However, each case followed a defined method, LQQTSFA combined with relevant guideline references, indicating a degree of reproducibility. Future research should aim to accumulate additional cases and evaluate educational outcomes to further verify the practical and instructional utility of structured symptom-based assessment in community pharmacy practice.

## Conclusions

This case series indicates that community pharmacists can support rational referral decisions, without engaging in diagnosis, by applying symptom-based assessment with the LQQTSFA framework and cross-checking findings against relevant clinical guidelines. Across three cases, this combination provided a reproducible basis for referral recommendations and pharmacy interventions, enabling scientifically grounded advice rather than reliance on subjective experience. Readily accessible guidelines and evidence also offered practical support for judging whether physician referral or self-medication management was appropriate. Although this approach does not replace diagnosis, it represents a pragmatic method to improve the quality of first-line responses in community pharmacies and to promote safe, efficient healthcare-seeking within community systems. Future work should assess reproducibility and generalizability through multicenter case accumulation and educational evaluations.
